# Altered Regional Homogeneity and Functional Connectivity in Patients with Painful Temporomandibular Disorders: A Multimodal Resting-state fMRI Study

**DOI:** 10.2174/0115734056480808260903070922

**Published:** 2026-09-22

**Authors:** Xin Li, Yujiao Jiang, Yuqi Deng, Zhiye Chen, Song Zhang

**Affiliations:** 1 Department of Nuclear Medicine, Xinqiao Hospital, Army Medical University, Chongqing 400037, China; 2 Department of Radiology, Hainan Hospital of PLA General Hospital, Sanya 572015, China

**Keywords:** Temporomandibular disorders, Pain, Resting-state functional MRI, Regional homogeneity, Enhancement of neuronal, Neuromodulation strategies, Functional connectivity

## Abstract

**Introduction:**

Although painful Temporomandibular Disorders (p-TMD) represent a common chronic pain condition, their central pathophysiology remains elusive. This work utilized multimodal resting-state functional MRI (rs-fMRI) to investigate functional brain alterations in patients with p-TMD.

**Methods:**

A total of 74 participants (41 p-TMD patients and 33 age- and gender-matched normal controls (NCs)) were enrolled in this case-control study. Each participant underwent both rs-fMRI and high-resolution structural imaging. We assessed multiple metrics, including Regional Homogeneity (ReHo), Amplitude of Low-Frequency Fluctuations (ALFF), Degree Centrality (DC), and further explored seed-based Functional Connectivity (FC). Intergroup differences were examined, and correlations between significant neuroimaging findings and clinical variables were assessed.

**Results:**

Relative to the NC group, p-TMD patients showed a specific increase in ReHo localized to the Left Postcentral Gyrus (L_PoG), with no concurrent changes in ALFF or DC in this region. This hyper-synchronous region demonstrated enhanced FC when the identified L_PoG cluster was used as the seed region in several brain regions, including the right caudate, left putamen, right postcentral gyrus, and right precentral gyrus. Notably, these altered ReHo and FC values showed no significant correlation with clinical variables.

**Discussion:**

The observed pattern of increased L_PoG ReHo in the absence of ALFF or DC changes suggests a highly localized enhancement of neuronal synchrony in the primary somatosensory cortex, rather than generalized hyperactivation or altered network hub status. The enhanced FC with the basal ganglia and contralateral sensorimotor cortex indicates aberrant integration of sensory and affective-motivational information. The lack of correlation with clinical measures suggests the brain alterations may better reflect stable disease traits than momentary pain states in p-TMD.

**Conclusion:**

The current study identified L_PoG neuronal hypersynchrony and enhanced sensorimotor-motivational network coupling as central features of p-TMD, which may be a manifestation of pain-related neuroplasticity. Collectively, this study provides a neuroimaging basis for developing new biomarkers and suggests potential for future targeted neuromodulation strategies in p-TMD.

## 1 INTRODUCTION

Temporomandibular Disorders (TMD) are frequently encountered in clinical dentistry, presenting with signs and symptoms that include orofacial pain, audible joint sounds, and restricted mandibular function. TMD represents a highly prevalent chronic pain condition, with an overall prevalence of approximately 31% in adults and 11% in children; however, TMD-related symptoms have been reported in as many as 50% of adults [1, 2]. Common precipitating factors include traumatic lesions, degenerative lesions of the Temporomandibular Joint (TMJ), and psychological stressors [3]. Painful TMD (p-TMD) often follows a prolonged course, causing pain and dysfunction in the TMJ region and showing high comorbidity with emotional disorders [4, 5]. This condition is a substantial public health issue, as it is the second most frequent musculoskeletal ailment, following chronic low back pain. Despite the substantial burden of p-TMD on quality of life, the underlying mechanisms of p-TMD remain to be fully elucidated.

The pathophysiological causes of p-TMD have previously been attributed mainly to peripheral factors, such as recurrent muscle tension or overload on the TMJ, which may increase the production of inflammatory mediators and neuropeptides that ultimately cause pain [6]. Nevertheless, persistent nociceptive input from peripheral sites is known to drive neuroplastic reorganization within central pain pathways, leading to heightened pain sensitivity [7]. Recent research has also indicated that p-TMD may share pathophysiological similarities with fibromyalgia and chronic lower back pain in relation to the central nervous system [8-10]. Therefore, understanding the mechanism of p-TMD requires further clarification of the functional alterations in the central nervous system.

Resting-state functional MRI (rs-fMRI) offers a powerful non-invasive tool for investigating central nervous system alterations through metrics such as Regional Homogeneity (ReHo), Amplitude of Low-Frequency Fluctuations (ALFF), Degree Centrality (DC), and Functional Connectivity (FC) [11]. These metrics capture complementary aspects of brain function [12-14]. Previous studies have demonstrated that TMD is associated with reduced FCs in corticostriatal networks and functional alterations in top-down pain modulatory and sensorimotor circuits [15, 16]. TMD patients have dysregulated spontaneous activity and FC in the default mode network, sensorimotor network, and pain-related regions [17]. This multimodal analytical framework, integrating local (ALFF, ReHo, DC) and network-level (FC) metrics, enables a comprehensive understanding of brain dysfunction in p-TMD patients. However, most previous studies employed single imaging metrics, and systematic multimodal characterization from local activity to network connectivity remains limited.

Based on these previous findings, we predicted that: (1) p-TMD patients would exhibit altered local brain activity (ReHo, ALFF, or DC) in sensorimotor regions; (2) such locally altered regions would show abnormal FC with affective-motivational circuits; and (3) these neural alterations might correlate with clinical pain or emotional measures. This study aimed to provide preliminary neuroimaging evidence for comprehending the central brain mechanisms underlying p-TMD by addressing these topics.

## 2 MATERIALS AND METHODS

### 2.1 Participants

This study received ethical approval from the Ethics Committee of the Hainan Hospital of PLA General Hospital (approval number: S2022-03), and all procedures were conducted in accordance with the Declaration of Helsinki. Written informed consent was obtained from every participant prior to enrollment.

A total of 74 right-handed participants aged 18-60 years were enrolled in this case-control study between October 2022 and January 2024. Forty-one p-TMD patients (M/F 18/23) were examined and diagnosed by an experienced specialist according to Diagnostic Criteria for Temporomandibular Disorders (DC/TMD), which represents the current standardized framework for TMD classification and assessment [18, 19]. The control group comprised 33 age- and gender-matched normal controls (NCs, M/F 12/21) with no history of chronic pain or major neurological disorders. The sample size was determined based on the exploratory nature of this study, which aimed to detect potential effect size differences in brain functional patterns. Although the sample size is relatively modest, it is within the range of those employed in previous exploratory rs-fMRI studies of TMD that have successfully identified meaningful neural alterations [20, 21].

Inclusion criteria for the p-TMD group were as follows: (1) presence of TMD-related pain affecting the TMJ, masticatory muscles, or craniofacial region, occurring either at rest or during function; (2) being in the interictal stage, defined as at least three days having elapsed since the most recent pain episode; (3) pain symptoms persisting for a minimum of three months, with no treatment received during the preceding two weeks. Exclusion criteria applied to all participants included: (1) psychiatric disorders; (2) history of temporomandibular joint pathologies; (3) metabolic disease; (4) MRI contraindications (*e.g*., metal implants, claustrophobia); (5) poor image quality.

Clinical characteristics of all subjects were assessed using Axis II of the DC/TMD, encompassing the Jaw Functional Limitation Scale (JFLS), the Patient Health Questionnaire-9 (PHQ-9) for depression, the Generalized Anxiety Disorder-7 (GAD-7) for anxiety, the Patient Health Questionnaire-15 (PHQ-15) for evaluation of somatization symptoms, and the Oral Behavior Checklist (OBC) for assessing the frequency of oral parafunctional behaviors. To ensure standardized psychiatric assessment, all subjects also completed the Hamilton Anxiety Scale (HAMA) and the Hamilton Depression Scale (HAMD) under the guidance of two psychiatrists. Pain intensity was quantified in the p-TMD group using a 0–10 Visual Analogue Scale (VAS), where 0 indicated no pain, and 10 represented the worst imaginable pain [22].

### 2.2 MRI Data Acquisition

MRI data acquisition was performed on a 3.0T MR system (Philips, Ingenia CX, Netherlands) with a 32-channel head coil. Participants were placed in the supine position, and foam pads were used to restrict head motion. During scanning, participants were instructed to stay awake, keep their eyes closed, relax, and remain still. The imaging protocol comprised the following sequences:

(1) High-resolution T1-weighted anatomical scan: A three-dimensional fast field echo (3D-FFE) sequence was used with the following parameters: Repetition Time/Echo Time (TR/TE) = 6.6/3.0 ms, matrix = 220 × 240, Flip Angle (FA) = 8°, Field of View (FOV) = 220 mm× 240 mm, slice thickness = 1 mm, slices =150, voxel size = 1 mm × 1 mm × 1 mm.

(2) rs-fMRI: bold signals were acquired using a Gradient-Echo Echo-Planar Imaging (GRE-EPI) sequence: TR/TE = 800/32 ms, matrix = 88 × 97, FA = 52°, FOV = 210 mm × 236 mm, slice thickness = 2 mm, Number of Excitations (NEX) = 1, voxel size = 2.4 mm × 2.4 mm × 2mm, no gap. A total of 180 volumes were collected, with whole-brain coverage including both cerebrum and cerebellum.

### 2.3 MRI Data Processing and Analysis

Preprocessing and statistical analysis of neuroimaging data were conducted using the RESTplus toolkit (v1.25) and Statistical Parametric Mapping software (SPM12, Wellcome Centre for Human Neuroimaging, London, UK) within the MATLAB 2021b environment (The MathWorks, Natick, MA, USA) [23].

Preprocessing of the rs-fMRI data followed a standardized pipeline comprising several sequential steps: (1) removal of the first 10 volumes; (2) slice timing correction; (3) realignment for head motion correction; (4) spatial normalization to the standard Montreal Neurological Institute (MNI) space using the deformation fields derived from segmenting the T1 image; (5) detrending; (6) nuisance covariate regression: including the white matter signal, cerebrospinal fluid signal and the 24-parameter model of head motion (Friston-24); (7) band-pass filtering (0.01–0.08 Hz).

ReHo, DC, and fALFF were calculated from the preprocessed bold time series and Z-score transformation. Based on the group-level findings from the multimodal analysis, the brain region exhibiting the most significant alteration in the p-TMD group was defined as the seed Region of Interest (ROI). This seed was then used to perform whole-brain FC analysis, in which Pearson's correlation coefficients were calculated between the averaged time series of the seed region and the time series of all other voxels across the brain. For each neuroimaging metric, group differences between p-TMD and NCs were assessed using a two-sample t-test within the general linear model framework in SPM12. Age and gender were included as nuisance covariates to control for potential confounding effects. To address the issue of multiple comparisons across the entire brain, Gaussian Random Field (GRF) theory was applied at the cluster level. The statistical threshold was set at a voxel-level significance of *P* < 0.001 and a cluster-level significance of *P* < 0.05 (GRF-corrected). For brain regions that did not survive the GRF correction, exploratory analyses were conducted using a more lenient threshold of *P* < 0.001 (uncorrected) at the voxel level combined with a cluster size threshold of *k* >20 voxels to minimize false positives, an approach commonly adopted in rs-fMRI studies [24, 25]. Anatomical localization of significant clusters was performed using the AAL90 atlas.

### 2.4 Statistical Analysis

Demographic and clinical data were processed using IBM SPSS 25 (SPSS, Chicago, Illinois, United States). Normally distributed continuous variables were expressed as mean ± standard deviation, whereas non-normally distributed variables were summarized as median with interquartile range (first quartile, third quartile). Independent two-sample t-tests were used for continuous variables, and chi-square tests were used for categorical variables to compare clinical data. Pearson's correlation coefficient was used for normally distributed data, and Spearman's rank correlation for non-normally distributed data. A significance threshold of *P* < 0.05 (two-tailed) was adopted for all statistical tests.

## 3 RESULTS

### 3.1 Demography and Clinical Characteristics

A detailed comparison of demographic and clinical variables between the p-TMD (n = 41) and NC (n = 33) groups is presented in Table **1**. Age, sex distribution, and assessments of psychological status, including scores on the HAMA, HAMD, GAD-7, PHQ-9, PHQ-15, and the OBC, were comparable between groups (all *P* > 0.05). As expected, individuals in the p-TMD group reported significantly greater impairment in jaw function, as reflected in notably higher JFLS scores than NCs (*P* < 0.05). The median pain intensity reported on the VAS was 3.00 (interquartile range: 2.00–4.50).

### 3.2 Altered Regional Homogeneity in p-TMD Patients

When compared with NCs, the p-TMD group exhibited increased ReHo values in the left postcentral gyrus (L_PoG) (peak MNI coordinates: −63, −18, 36; cluster size = 28; *P_unc_* < 0.001; cluster > 20), as detailed in Table **2** and illustrated in Fig. (**1**). Conversely, no brain regions showed significantly reduced ReHo in the p-TMD group relative to NCs. In contrast, analyses of fALFF and DC did not reveal any significant group differences across the whole brain.

Partial correlation analyses found no significant associations between the mean ReHo value extracted from the significant L_PoG cluster and any clinical variables in the p-TMD group (all *P* > 0.05).

### 3.3 Altered Functional Connectivity Value with the L_PoG Seed in p-TMD Patients

Using the statistically different brain area as a seed point, FC analysis was subsequently conducted with the identified L_PoG cluster as the seed region. Compared with NCs, p-TMD patients exhibited significantly enhanced FC between the L_PoG seed and several brain regions, including the right caudate, left putamen, right postcentral gyrus (R_PoG), and right precentral gyrus (R_ PrG) (Table **3**, Figs. **2** and **3**). In contrast, no clusters showed reduced FC with the L_PoG seed in the patient group.

Similar to the ReHo findings, further exploratory analyses did not identify any statistically significant correlations between the mean FC strength of these four altered pathways and the clinical parameters within the p-TMD cohort (all *P* > 0.05).

## 4 DISCUSSION

Through a multimodal rs-fMRI analysis, this study aimed to clarify the core brain mechanisms underlying p-TMD. We observed a pattern of functional brain changes characterized by dysregulation at both local and network levels. Specifically, p-TMD patients exhibited significantly increased ReHo in the L_PoG and increased FC with two key regions: (1) the contralateral sensorimotor cortex and (2) subcortical nuclei of the basal ganglia. Notably, these neural alterations did not correlate with clinical pain or emotion scores. These findings suggest that p-TMD is a central nervous system disorder involving functional alterations in specific brain networks, rather than solely a peripheral joint disease.

The primary somatosensory cortex, located in the postcentral gyrus, processes various sensory modalities, including the discriminative aspects of nociception. In the absence of ALFF or DC alterations, the isolated increase in ReHo of the L_PoG suggests a highly localized modification. As each metric reflects a fundamentally different aspect of brain function, this dissociation is notable. This pattern suggested that chronic nociceptive input from the temporomandibular region did not lead to generalized activation of the homologous cortex (as measured by ALFF), nor did it reposition this region as a major hub within the broader pain network (as measured by DC) [26]. Rather, it appears to reflect a specific enhancement in the local temporal synchronization of neuronal ensembles processing orofacial sensation [13]. Therefore, changes in ReHo may be more sensitive than other indicators to anomalies in the fine temporal organization of sensory processing in p-TMD. This finding is consistent with growing evidence that chronic pain is associated with condition-specific brain alterations. In contrast to individuals with irritable bowel syndrome, TMD patients showed a distinct ReHo increase in the left supplementary motor region, indicating that visceral and somatic pain may share abnormal activity in multiple brain networks [27]. This suggests distinct local functional reorganization patterns for various chronic pain conditions. However, the current investigation identified elevated ReHo only within the L_PoG among p-TMD patients, which may be explained by hemispheric lateralization of pain processing, with the left hemisphere preferentially engaged in sustained attention to pain and its affective appraisal [28]. Furthermore, the dominant hemisphere’s somatosensory cortex may undergo more pronounced neuroplastic reorganization [29]. Consequently, the brain's adaptive response to ongoing pain signals from the TMJ region may underlie the observed localized hypersynchrony in the L_PoG [30]. This change most likely serves as a crucial neuronal basis for the long-lasting sensory impairments that p-TMD patients experience. Future studies with systematic pain laterality assessment are needed to further clarify this pattern.

Subsequently, the L_PoG was found to exhibit enhanced functional connectivity with the basal ganglia and the contralateral somatosensory cortex, suggesting altered integration of sensory and affective-motivational information in p-TMD. Reduced cortical thickness in the right sensorimotor cortex in TMD patients was reported in earlier research, indicating a significant involvement of the sensorimotor cortex in the pathophysiology of chronic pain [20]. Furthermore, the brain alterations associated with p-TMD in the current study involved bidirectional functional network integration, rather than only unilateral cortical function abnormalities. This may reflect the fact that chronic pain can produce aberrant bilateral somatosensory cortex connections, ultimately resulting in alterations in neuroplasticity [6]. The increased L_PoG ReHo and its enhanced FC with the basal ganglia and the contralateral sensorimotor cortex may serve as potential neuroimaging biomarkers for central dysfunction in p-TMD, aiding differential diagnosis and patient selection for centrally targeted therapies.

Traditionally regarded as central hubs integrating sensation, emotion, and motivated behavior, the basal ganglia are increasingly recognized for their role in chronic pain [31, 32]. The current study's finding of enhanced connectivity between the L_PoG and basal ganglia demonstrates a strong association between impaired sensory processing and emotional processing centers in chronic pain. Previous research has shown altered functional connectivity and anatomical changes in the putamen, highlighting the crucial role of the basal ganglia in TMD patients [20, 33, 34]. Persistent pain may lead to additional anatomical and functional issues in the brain, potentially due to neural tissue damage, changes in extracellular matrix volume, or even atrophy [35]. As a result, impulses related to persistent orofacial pain may be inappropriately routed into circuits that regulate emotion generation. The L_PoG–basal ganglia pathway may explain the comorbidity of p-TMD with negative emotions such as anxiety and depression [36, 37].

The current correlation analysis revealed no statistically significant association between clinical variables (*e.g*., VAS) and brain alterations (increased L_PoG ReHo and its enhanced FC) (*P*-value > 0.05). This dissociation suggests that the brain metrics may better reflect stable disease traits than momentary pain states in p-TMD. This may be because alterations in specific brain circuits may not be directly reflected in the clinical scales, which measured a general subjective state. Future studies should therefore focus on determining whether these changes can predict long-term outcomes or guide specific p-TMD therapies.

## 5 STUDY LIMITATIONS

Several limitations of the present work should be acknowledged. First, it was a cross-sectional observation, necessitating prospective longitudinal studies to track neural changes over time. Second, additional multimodal MR techniques, such as magnetic resonance spectroscopy and perfusion-weighted imaging, were not employed. Third, the relatively modest sample size, resulting from strict enrollment criteria, restricted our capacity to perform subgroup analyses; therefore, large-sample studies are required to validate and extend our findings.

## CONCLUSION

In conclusion, the current rs-fMRI analysis revealed two key characteristics of p-TMD: L_PoG neuronal hypersynchrony and increased sensorimotor-motivational network coupling. These changes, which define this condition as a distinct dysfunction of the brain network, may reflect neuroplasticity associated with pain. Thus, our work provides a neuroimaging basis for the development of novel biomarkers and highlights possibilities for future targeted neuromodulation strategies in p-TMD.

## Figures and Tables

**Fig. (1):**
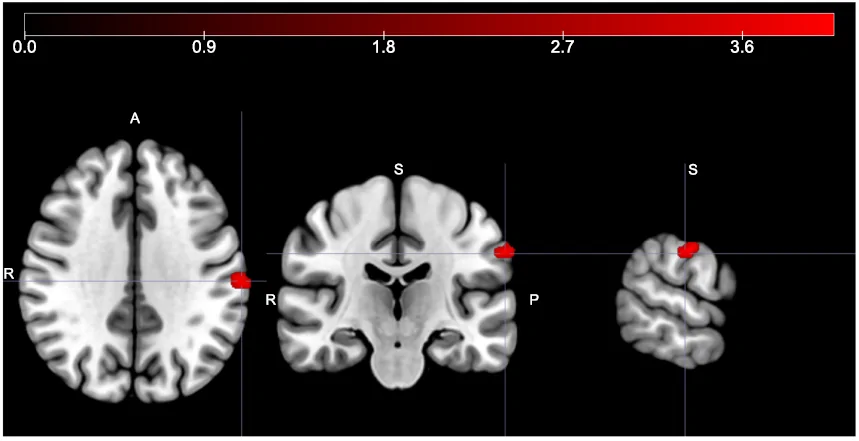
Brain region (L_ PoG) showing increased ReHo value in the p-TMD patients compared with NCs. The color bar represents the t-value. **Abbreviations:** L_PoG: Left Postcentral Gyrus.

**Fig. (2):**
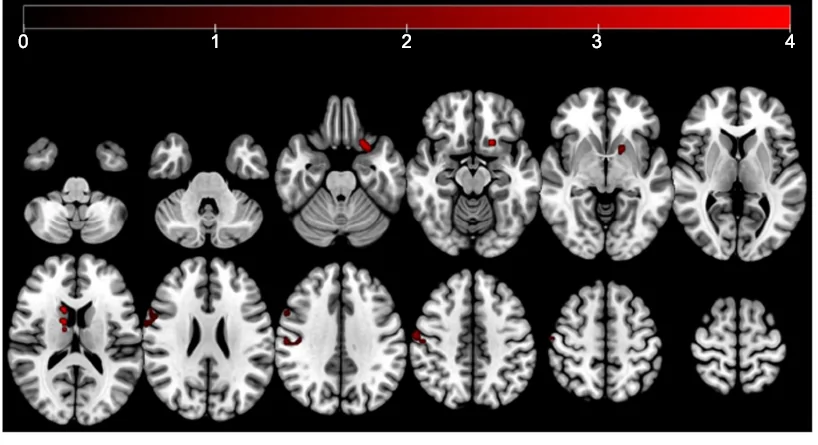
Enhanced functional connectivity of the L_PoG seed in p-TMD patients.

**Fig. (3):**
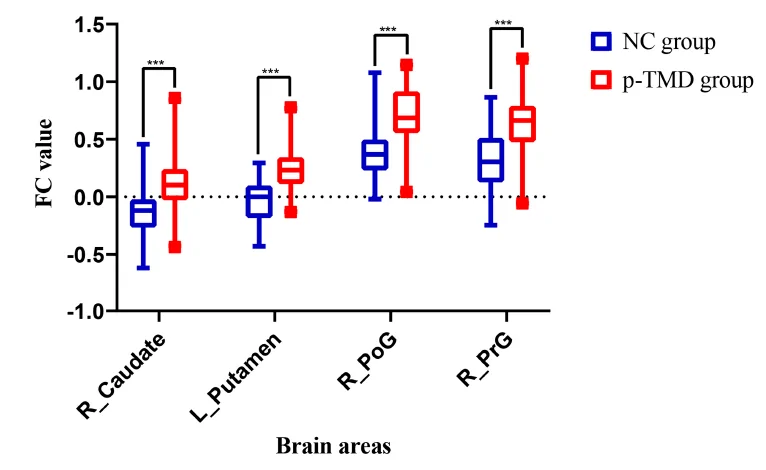
Box plots comparing the mean FC value between the L_PoG and each of the four target regions. ***: *p* < 0.001. **Abbreviations:** L_PoG: Left Postcentral Gyrus; R_Caudate: Right Caudate; L_Putamen: Left Putamen; R_PoG: Right Postcentral gyrus; R_PrG: Right Precentral Gyrus.

**Table 1:** Demographic and clinical assessments of the participants.

-	p-TMD	NCs	*P*-value
Num(M/F)	41(18/23)	33(12/21)	0.511
Age^a^	25.00(33.00, 22.00)	25.00(26.50, 23.00)	0.785
HAMA^a^	4.00(6.00, 2.00)	3.00(6.50, 1.00)	0.450
HAMD^a^	3.00(5.00, 1.00)	2.00(4.00, 1.00)	0.305
GAD-7^a^	3.00(7.50, 0.00)	1.00(5.00, 0.00)	0.157
PHQ-9^a^	4.00(8.50, 1.00)	4.00(7.00, 2.00)	0.710
PHQ-15^a^	3.00(8.50, 0.50)	3.00(5.00, 0.00)	0.353
JFLS^a^	35.00(50.00, 16.00)	3.00(6.50, 0.00)	0.000
OBC^a^	18.00(24.50, 13.50)	15.00(26.00, 11.50)	0.439
VAS^a^	3.00(4.50, 2.00)	NA	NA

**Abbreviations:** p-TMD, Painful Temporomandibular Disorders; NCs, Normal Controls; HAMA: Hamilton Anxiety Scale; HAMD: Hamilton Depression Scale; GAD-7: Generalized Anxiety Disorder-7; PHQ-9: Patient Health Questionnaire-9; PHQ-15: Patient Health Questionnaire-15; JFLS: Jaw Functional Limitation Scale; OBC: Oral Behavior Checklist; VAS: Visual Analogue Scale; NA: Not Available; ^a^Data are presented as Median (the first quartile, the third quartile).

**Table 2:** Brain region showing increased ReHo in p-TMD group.

**Anatomic Region**	**MNI-space**			**Cluster Size**	***P*_uncorr_**	**Peak T-value**
	**X**	**Y**	**Z**			
L_PoG	-63	-18	36	28	0.000	4.10

**Note:** Statistical threshold was set at voxel-level *P* < 0.001 (uncorrected) with a cluster size > 20 voxels. MNI, Montreal Neurological Institute.

**Abbreviation:** L_PoG: Left Postcentral Gyrus.

**Table 3:** Brain regions showing altered functional connectivity with the L_ PoG seed in p-TMD patients.

**Anatomic Region**	**MNI-space**			**Cluster Size**	***P*_uncorr_**	**Peak T-value**
	**X**	**Y**	**Z**			
R_Caudate	12	-3	18	24	0.000	4.57
L_Putamen	-15	9	-6	44	0.000	4.40
R_PoG	60	-12	48	50	0.000	4.38
R_PrG	57	12	30	59	0.000	5.15

**Note:** Statistical threshold was set at voxel-level *P* < 0.001 (uncorrected) with a cluster size > 20 voxels.

**Abbreviations:** R_Caudate: Right Caudate; L_Putamen: Left Putamen; R_PoG: Right Postcentral gyrus; R_PrG: Right Precentral gyrus; MNI, Montreal Neurological Institute.

## Data Availability

The authors confirm that the data supporting the findings of this research are available within the article.

## LIST OF ABBREVIATIONS

ALFF: Amplitude of Low-Frequency Fluctuations
DC: Degree Centrality
FC: Functional Connectivity
GAD-7: Generalized Anxiety Disorder-7
HAMA: Hamilton Anxiety Scale
HAMD: Hamilton Depression Scale
JFLS: Jaw Functional Limitation Scale
MNI: Montreal Neurological Institute
NA: Not Available
NCs: Normal Controls
OBC: Oral Behavior Checklist
p-TMD: Painful Temporomandibular Disorders
PHQ-9: Patient Health Questionnaire-9
PHQ-15: Patient Health Questionnaire-15
L_Putamen: Left Putamen
R_Caudate: Right Caudate
ReHo: Regional Homogeneity
R_PoG: Right Postcentral Gyrus
R_PrG: Right Precentral Gyrus
rs-fMRI: Resting-state Functional Magnetic Resonance Imaging
VAS: Visual Analogue Scale

## STANDARDS OF REPORTING

STROBE guidelines were followed.
